# A PATH ANALYSIS OF THE HEALTHCARE UTILIZATION AND SERVICES SATISFACTION AMONG COMMUNITY-DWELLING INDIVIDUALS WITH SPINAL CORD INJURY IN MALAYSIA

**DOI:** 10.2340/jrm.v57.40621

**Published:** 2025-02-25

**Authors:** Muhamad F. ZAINUDIN, Natiara M. HASHIM, Wan N.W.M. ZOHDI, Nazirah HASNAN, Julia P. ENGKASAN

**Affiliations:** 1 Department of Rehabilitation Medicine, Faculty of Medicine, Universiti Teknologi MARA, Sungai Buloh; 2 Department of Rehabilitation Medicine, Faculty of Medicine, Universiti Malaya, Kuala Lumpur, Malaysia

**Keywords:** spinal cord injury, rehabilitation, healthcare, health services, patient satisfaction

## Abstract

**Purpose:**

To explore healthcare utilization patterns and healthcare services satisfaction among individuals with spinal cord injury in Malaysia.

**Methods:**

This cross-sectional study utilized the International Spinal Cord Injury (InSCI) Community Survey and involved 8 hospitals and 1 spinal cord injury organization. A total of 285 participants met the inclusion criteria. Subsequently, 6/11 sections of the InSCI questionnaire were analysed through a path analysis.

**Results:**

The 3 most utilized healthcare providers reported were physical and rehabilitation medicine specialists (76.5%), physiotherapists (36.8%), and primary care physicians (27.4%). The top 3 most severe health problems reported were sexual dysfunction, muscle spasm and spasticity, and contractures. Healthcare services satisfaction was high. Health problems predicted healthcare utilization (β = 0.443), while activity limitation and participation restriction predicted healthcare services satisfaction (β = –0.202). The activity limitation and participation restriction in male participants was moderated by the spinal cord injury severity (B = 2.330, *p* < 0.001) and health problems (B = 0.550, *p* < 0.001).

**Conclusions:**

Individuals with spinal cord injury in Malaysia rely heavily on physical and rehabilitation medicine specialists, highlighting accessibility challenges due to the centralized specialized rehabilitation services. Sexual dysfunction remains a significant yet under-addressed health concern. Despite these issues, satisfaction with healthcare services is high.

## LAY ABSTRACT

This study looked at how people with spinal cord injuries in Malaysia use healthcare services and how satisfied they are with these services. We surveyed 285 people from 8 hospitals and 1 spinal cord injuries organization across different parts of the country. The most common healthcare providers they visited were specialists in rehabilitation, physiotherapists, and primary care doctors. Participants said that sexual problems, muscle spasms, and stiffness were their most significant health issues. The study found that health problems affected how often people with spinal cord injuries used healthcare services, while their ability to do daily activities influenced their satisfaction. Overall, people with spinal cord injuries in Malaysia were quite satisfied with the healthcare services.

Studies indicate that individuals with spinal cord injuries (SCI) have greater contact rates with the health system than the general population (1, 2). They have a median of 22 contact points in the year of injury compared with just 3 for the general population (2). Hospital readmission rates peak in the first 5 years following injury, and secondary complications continue to affect individuals with SCI long after the initial trauma (2). Poor satisfaction with healthcare is strongly related to long travel time to SCI centres and lack of access to suitable transportation (3). Despite their complex needs and higher rates of healthcare utilization (HCU), individuals with SCI continue to face barriers when accessing healthcare resources (1).

Malaysia, comprising 13 states and 3 federal territories, is divided into 5 regions: Central, Northern, Southern, East Coast, and East Malaysia. The Malaysian healthcare system features a dual-tiered service: a highly subsidized publicly funded sector and a fee-for-service private sector, creating a dichotomous public–private model (4, 5). Although the sustainability of this 2-tiered system is debatable, it has greatly improved the population’s health and transformed the country into one of the healthiest in the tropics (5). The increase in life expectancy from 63.1 years (male) and 66.0 years (female) in 1966 to 72.6 and 77.2 years in 2010, respectively, in addition to improved neonatal, child, and maternal mortality rates, indicates better health outcomes (5). Malaysia spends only 4–5% of its gross domestic product on healthcare (6).

Approximately 10 million Malaysians, out of a total population of 32.7 million (7), could benefit from rehabilitation services (8). Physical and rehabilitation medicine (PRM), as a medical discipline, is relatively nascent in the country, with its first batch of home-grown PRM specialists graduating in 2001. For 2 decades, just 1 training centre offered the residency programme, resulting in a modest output of fewer than 120 PRM specialists since its inception. Despite these humble beginnings, the fraternity has broadened its reach across all 5 regions of Malaysia. Specialized rehabilitation services led by PRM specialists are now available in all state hospitals except Perlis, the smallest state in the Northern region. However, the distribution is uneven, with the Central region holding the majority of PRM specialists, posing accessibility issues.

Previous research on SCI rehabilitation in Malaysia has predominantly focused on domains such as body structure and functions (9–11), activity limitation (12, 13), and participation restriction (14–17) of the International Classification of Functioning, Disability and Health (ICF), with little emphasis on HCU or healthcare services satisfaction (HCSS). Furthermore, these studies were mainly single-centred and conducted in urbanized regions, limiting their generalizability. Our study explored the patterns of HCU and HCSS and the predictors influencing these aspects among community-dwelling individuals with SCI in Malaysia. Drawing comparisons with international findings is crucial for understanding the local facilitators and barriers to HCU and the factors influencing patients’ satisfaction with rehabilitation services. Given Malaysia’s unique blend of public–private healthcare models and the centralized specialized rehabilitation services, it is critical to identify gaps and refine service delivery. The findings could offer insights for policymakers and stakeholders in the provision of more accessible and inclusive healthcare services for individuals with SCI and people with disabilities (PWDs) at large. This study also highlights potential gaps in the current healthcare system to guide future clinical service improvement and rehabilitation research in Malaysia.

## METHODS

### Study design and sample size

This cross-sectional study was conducted as part of the International Spinal Cord Injury Community Survey (InSCI) project involving 28 countries (18). In Malaysia, the Department of Rehabilitation Medicine, Universiti Malaya, coordinated this study in collaboration with 8 hospitals and an SCI organization spanning the 5 Malaysian economic regions. The participating institutions were the Universiti Malaya Medical Centre, Cheras Rehabilitation Hospital, and Sungai Buloh Hospital from the Central region, Penang and Ipoh Hospital from the Northern region, Johor Bahru Hospital from the Southern region, Kota Kinabalu and Kuching Hospital from East Malaysia, and the East Coast Spinal Injury Association from the Eastern region. A minimum sample size of 200 participants was determined based on a power analysis using the Swiss SCI (SwiSCI) community survey data (18). The Malaysian InSCI national study protocol is described elsewhere (19).

### Participants and recruitment

The study was conducted over 17 months, from March 2017 to July 2018. Participants were recruited using a convenience sampling method by inviting individuals who attended SCI rehabilitation clinics at participating institutions and members of a local SCI organization. Data collection was conducted through a combination of paper–pencil surveys and face-to-face interviews. Inclusion criteria were traumatic and non-traumatic SCI, age 18 years or older, injury duration of more than 1 year, and community dwellers who reside in Malaysia. Exclusion criteria were congenital, progressive, and neurodegenerative diseases of the spine and those with cognitive impairment.

### Research instrument and measures

InSCI is a multinational community survey constructed based on the ICF Core Sets for SCI (18). It is designed to support the Learning Health System for SCI initiative embedded in the World Health Organization’s Global Disability Plan through the collection of internationally comparable data on the lived experience of persons with SCI (20). The questionnaire contains 125 questions, divided into 11 sections, covering 47 ICF categories (18). The English version was translated into Malay and Mandarin languages for Malaysian use. This report analysed data from 6 sections: personal information, lesion characteristics, health problems, activity and participation (reported as activity limitation and participation restriction [ALPR]), independence in activities of daily living (ADL), and healthcare services. Health problems, ALPR, and HCSS were assessed using a 5-point Likert scale, while independence in ADL was measured using variable Likert scales ranging from 2 to 6 points. For health problems and ALPR, a score of 1 indicated no problem, while a score of 5 indicated extreme problems. For items concerning HCSS, a score of 1 indicated very bad or very dissatisfied, and a score of 5 indicated very good or very satisfied. For independence in ADL, the lowest score indicated total assistance, while the highest score indicated total independence. The variables were analysed as they were categorized in the questionnaire, except for education level, which was classified as either low (primary and secondary education) or high (post-secondary education), in accordance with the Malaysian Qualifications Framework (21). Further details on the questionnaire development are described elsewhere (18).

### Statistical analysis

The statistical analysis was conducted sequentially using the Statistical Package for the Social Sciences (SPSS) version 23 (IBM Corp, Armonk, NY, USA). First, descriptive analysis was performed on demographic and independent variables, expressing categorical data in frequencies and percentages. Continuous variables, including health problems, ALPR, independence in ADL, and HCSS, were reported in overall means with standard deviations. Additionally, the means of individual components of health problems were analysed separately for more detailed insights. Then, a Pearson’s correlation analysis was conducted to validate the inclusion of multiple variables in the model and to prevent biased estimations due to collinearity (22). An acceptable correlation coefficient between 2 independent variables was set to be between –0.7 and 0.7.

Subsequently, path analysis was performed using SPSS Amos to model the complex causal relationships between variables. Path analysis, an extension of multiple regression, enables the analysis of more complicated models beyond regular regression analysis, such as those with multiple dependent variables or those involving chains of influence between variables (23). The model’s appropriateness was first evaluated using 5 measures: root mean square error of approximation (RMSEA), χ^2^/degree of freedom, comparative fit index (CFI), Tucker–Lewis index (TLI), and incremental fit index (IFI). Standardized beta coefficients were applied to estimate the relationship’s direction and magnitude between variables. A mediation analysis was then performed to examine any indirect effects within the model. To conclude, a 2-group analysis was conducted to explore any moderating effects of gender differences in the model.

## RESULTS

### Descriptive analysis

Of 298 respondents who participated in the survey, 13 were excluded due to missing data, leaving 285 participants for the final analysis. In this study, most patients were male (78.9%), married (46.3%), had paraplegia (69.4%), were from low education levels (68.4%), and required assistance in day-to-day activities (78.2%). In terms of independent variables, the participants had a moderate mean of health problems (30.02, max: 62.00), a moderate mean of ALPR (42.65, max: 90.00), and a moderately high mean of independence in ADL (44.31, max: 64.00). The HCSS was high (mean 17.17, max: 20.00). Table I indicates the descriptive analysis.

**Table I:** Descriptive analysis of the demographics and independent variables (*n* = 285)

Demographic characteristics	*n* (%)	Mean (SD)
Gender		
Male	225 (78.9)	
Female	60 (21.1)	
Marital status		
Single	129 (45.3)	
Married	132 (46.3)	
Cohabiting	8 (2.8)	
Separated or divorced	14 (4.9)	
Widowed	2 (0.7)	
Education levels		
Low education (primary or secondary)	195 (68.4)	
High education (post-secondary)	90 (31.6)	
Assistance in day-to-day activities	233 (78.2)	
Family	202 (70.9)	
Friends	41 (14.4)	
Professionals or paid assistants	15 (5.3)	
Measures		
SCI severity		
Paraplegia incomplete	111 (38.9)	
Paraplegia complete	87 (30.5)	
Tetraplegia incomplete	56 (19.6)	
Tetraplegia complete	31 (10.9)	
Health problems		30.02 (8.56)
Independence in ADL		44.31 (10.76)
Activity limitation/participation Restriction		42.65 (14.09)
Health care services satisfaction		17.17 (2.07)

ADL: activities of daily living; SCI: spinal cord injury.

### Health problems

The top 5 most severe health problems reported were sexual dysfunction (mean 3.074, SD 1.445), muscle spasm and spasticity (mean 2.856, SD 1.277), contractures (mean 2.690, SD 1.301), pain (mean 2.528, SD 1.218), and bladder dysfunction (mean 2.507, SD 1.387) (Table II).

**Table II:** Health problems in spinal cord injury

Health problems	Mean (SD)
Sexual dysfunction	3.074 (1.445)
Muscle spasms, spasticity	2.856 (1.277)
Contractures	2.690 (1.301)
Pain	2.528 (1.218)
Bladder dysfunction	2.507 (1.387)
Bowel dysfunction	2.363 (1.256)
Sleep problems	2.233 (1.111)
Urinary tract infections	1.870 (1.126)
Pressure sores, decubitus	1.850 (1.234)
Circulatory problems	1.669 (1.047)
Injury caused by loss of sensation	1.660 (1.135)
Respiratory problems	1.585 (0.975)
Postural hypotension	1.576 (0.919)
Autonomic dysreflexia	1.563 (0.920)

### Healthcare utilization

The 3 most utilized healthcare providers reported were PRM specialists (76.5%), physiotherapists (36.8%), and primary care physicians (PCPs) (27.4%), while the 3 least utilized healthcare providers were psychologists (5.3%), home healthcare workers (3.5%), and chiropractors (0.4%) (Table III).

**Table III:** Healthcare providers visited in the last 12 months (Healthcare Utilisation)

Healthcare providers	*n* (%)
Rehabilitation physician/spinal cord injury physician	218 (76.5)
Physiotherapist	105 (36.8)
Primary care physician/general practitioner	78 (27.4)
Pharmacist	70 (24.6)
Occupational therapist	68 (23.9)
Nurse or midwife	56 (19.6)
Alternative medicine practitioner, e.g., naturopath, acupuncturist	53 (18.6)
Another specialist physician, e.g., surgeon, gynaecologist, psychiatrist, ophthalmologist	49 (17.2)
Dentist	39 (13.7)
Psychologist	15 (5.3)
Home healthcare worker	10 (3.5)
Chiropractor	1 (0.4)

### Correlational analysis

The Pearson correlation coefficients ranged from –0.676 to 0.507, which were within the acceptable range (Table IV). Hence, all variables were included in the path analysis.

**Table IV:** Correlational analysis (*n* = 285)

Factor	SCI severity	Health problems	Independence in ADL	ALPR	Healthcare utilization	Healthcare services satisfaction
*r* (*p*-values)						
SCI Severity	1					
Health problems	0.150^*^ (0.011)	1				
Independence in ADL	0.375^**^ (0.000)	0.507^**^ (0.000)	1			
ALPR	–0.315^**^ (0.000)	–0.278^**^ (0.000)	–0.676^**^ (0.000)	1		
Healthcare utilisation	0.117^*^ (0.048)	0.483^**^ (0.000)	–0.167^**^ (0.005)	0.301^**^ (0.000)	1	
Healthcare services satisfaction	0.069 (0.246)	0.151^*^ (0.011)	–0.118^*^ (0.047)	0.208^**^ (0.000)	–0.011 (0.857)	1

* Correlation is significant at the 0.05 level (2-tailed).

** Correlation is significant at the 0.01 level (2-tailed).

ADL: activities of daily living; ALPR: activity limitation/participation restriction; SCI: spinal cord injury.

### Path analysis

**Fig. 1:**
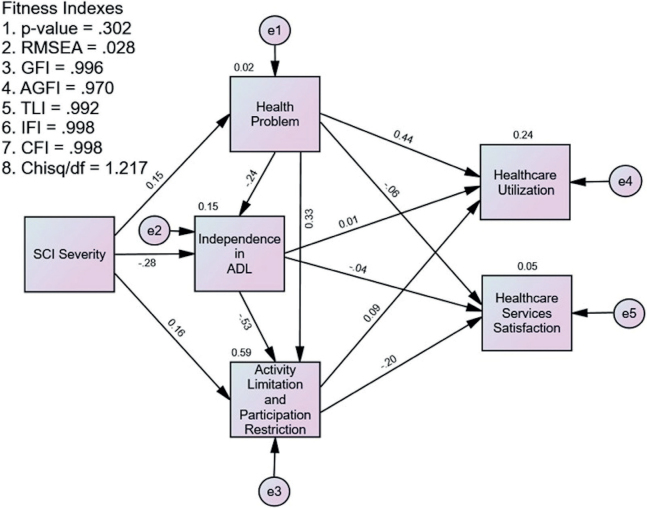
Statistical framework for the path analysis of the healthcare utilization and healthcare services satisfaction. ADL: activities of daily living; SCI: spinal cord injury.

The path analysis model achieved acceptable fit indices with (*i*) RMSEA: 0.028, (*ii*) χ^2^/degree of freedom: 1.217, (*iii*) CFI: 0.998, (*iv*) TLI: 0.992, and (v) IFI: 0.998. Fig. 1 illustrates the statistical framework, while Table V demonstrates the direct effects of all variables within the path analysis model. First, health problems were significantly predicted by the SCI severity (β = 0.150). The higher the SCI severity, the more severe the health problems. Second, independence in ADL was significantly predicted by the SCI severity and health problems (β = –0.279 and –0.237, respectively). The higher the SCI severity and the more severe the health problems, the lower the independence in ADL. Similarly, the ALPR was significantly predicted by SCI severity, health problems, and independence in ADL (β = 0.157, 0.335, and –0.534, respectively). The higher the SCI severity and health problems, the higher the ALPR, while the higher the independence in ADL, the lower the ALPR. Health problems significantly predicted the HCU (β = 0.443). The more severe the health problems, the higher the HCU. The independence in ADL or ALPR did not significantly predict the HCU. HCSS was only significantly predicted by ALPR (β = –0.202). The lower the ALPR, the higher the HCSS.

**Table V:** Direct effect of variables in the path analysis model

Hypothesized relationships	β	*t*-values (CR)	Hypothesis supported
H1: Spinal cord injury Severity → Health problems	0.150	2.557	Supported
H2: Spinal cord injury Severity → Independence in ADL	–0.279	–5.059	Supported
H3: Spinal cord injury Severity → ALPR	0.157	3.913	Supported
H4: Health problems → Independence in ADL	–0.237	–4.824	Supported
H5: Health problems → ALPR	0.335	8.437	Supported
H6: Health problems → Healthcare utilization	0.443	7.337	Supported
H7: Health problems → Healthcare services Satisfaction	–0.058	–0.860	Not supported
H8: Independence in ADL → ALPR	–0.534	–12.910	Supported
H9: Independence in ADL → Healthcare utilization	0.015	0.205	Not supported
H10: Independence in ADL → Healthcare services satisfaction	–0.035	–0.445	Not supported
H11: ALPR → Healthcare utilization	0.086	1.090	Not supported
H12: ALPR → Healthcare services satisfaction	–0.202	–2.295	Supported
Squared multiple correlation (R^2^)			
Health problems	0.023		
Independence in ADL	0.154		
ALPR	0.589		
Healthcare utilization	0.237		
Healthcare services satisfaction	0.047		
Model fit statistics			
RMSEA: 0.028, χ^2^/degree of freedom: 1.217, CFI: 0.998, TLI: 0.992, IFI: 0.998			

β: standardized estimate, CR: critical ratio, RMSEA: root mean square error of approximation, CFI: comparative fit index, TLI: Tucker–Lewis index, IFI: incremental fit index; ADL: activities of daily living; ALPR: activity limitation/participation restriction.

### Mediation analysis

Table VI depicts the mediation analysis in the path analysis model. The health problems completely mediated the direct effect of SCI severity and HCU but not independence in ADL or ALPR. The ALPR completely mediated the direct effect of SCI severity and HCSS, but not health problems and independence in ADL. The direct effect of health problems and ALPR was partially mediated by independence in ADL.

**Table VI:** Mediation analysis using a 5,000 bootstrap

Relationship	Direct effect (*t*-value)	*p*-value	Indirect effect	CI (95%)		*p*-value	Conclusion
				Low	High		
SCI severity → Health problems → HCU	0.099 (0.485)	0.628	0.242	0.088	0.423	0.010	Complete mediation
SCI severity → Independence in ADL → HCU			–0.018	–0.135	0.106	0.812	No mediation
SCI severity → ALPR → HCU			0.044	–0.027	0.137	0.287	No mediation
SCI severity → Health problems → HCSS	0.011 (0.085)	0.932	–0.018	–0.067	0.011	0.276	No mediation
SCI severity → Independence in ADL → HCSS			0.020	–0.049	0.102	0.613	No mediation
SCI severity → ALPR → HCSS			–0.066	–0.135	–0.020	0.016	Complete mediation
Health problems → Independence in ADL → ALPR	0.551 (8.437)	< 0.001	0.208	0.124	0.291	< 0.001	Partial mediation

ADL: activities of daily living; ALPR: activity limitation/participation restriction; HCSS: healthcare services satisfaction; HCU: healthcare utilization; SCI: spinal cord injury.

### Split-gender moderation analysis

The 2-group analysis (Table VII) achieved configural and metric invariance, making it eligible for moderation testing. The relationships were not moderated by gender except for the relationship between SCI severity and health problems with ALPR. First, the higher the SCI severity of male participants, the higher the ALPR (B = 2.330, *p* < 0.001), while the SCI severity did not significantly affect ALPR in female participants (B = 2.391, *p* = 0.103). Second, the more severe the health problems of male participants, the higher the ALPR (B = 0.550, *p* < 0.001), while the severity of health problems did not significantly affect ALPR in female participants (B = 0.492, *p* = 0.001).

**Table VII:** Split-gender moderation analysis

Hypothesized relationships	Male (*n* = 225)	Female (*n* = 60)	Group differences
	Estimates (*t*-values)		
H1: SCI Severity → Health problems	1.506 (2.663)	0.580 (0.560)	0.613
H2: SCI Severity → Independence in ADL	–1.989 (–2.965)	–5.564 (–4.948)	0.001
H3: SCI severity → ALPR	2.330 (3.900)	2.391 (1.629)	7.198
H4: Health problems → Independence in ADL	–0.396 (–5.074)	–0.017 (–0.121)	0.118
H5: Health problems → ALPR	0.550 (7.633)	0.492 (3.191)	5.435
H6: Health problems → HCU	0.188 (6.281)	0.185 (3.533)	0.003
H7: Health problems → HCSS	–0.016 (–0.800)	–0.014 (–0.490)	0.002
H8: Independence in ADL → ALPR	–0.746 (–12.759)	–0.528 (–3.711)	1.972
H9: Independence in ADL → HCU	–0.023 (–0.804)	0.067 (1.461)	1.166
H10: Independence in ADL → HCSS	–0.005 (–0.245)	–0.011 (–0.424)	0.171
H11: ALPR → HCU	0.007 (0.277)	0.057 (1.430)	2.738
H12: ALPR → HCSS	–0.026 (–1.669)	–0.038 (–1.695)	0.039
Model fit across the groups:			
RMSEA: 0.000, χ^2^/degree of freedom: 0.662, CFI: 1.000, TLI: 1.026, IFI: 1.006.			

ADL: activities of daily living; ALPR: activity limitation/participation restriction; HCSS: healthcare services satisfaction; HCU: healthcare utilization; SCI: spinal cord injury.

## DISCUSSION

This study is the first in Malaysia to successfully employ a path analysis model in investigating HCU and HCSS among individuals with SCI. Our analysis yielded 2 significant findings. First, the HCU was predicted by health problems, which in turn was predicted by the SCI severity. This suggests an influencing chain where more severe SCI gives rise to more serious health problems, leading to higher HCU and vice versa. Second, HCSS was predicted by ALPR, which in turn was predicted by the SCI severity. In this path, a more severe SCI results in greater ALPR, leading to lower HCSS and vice versa.

Our investigation of the HCU pattern revealed PRM specialists as the most utilized healthcare providers reported among community-dwelling individuals with SCI in Malaysia (78%), followed by physiotherapists (36.8%) and PCPs (27.4%). The HCU was based on the self-report frequency of visits to healthcare providers within the last 12 months. This trend was mirrored in Thailand (24) but contrasted with many countries in North America and Europe, where PCPs were the predominant healthcare providers for individuals with SCI (1, 2, 25–27). Interestingly, a cluster analysis of the InSCI data revealed 9 different HCU patterns, irrespective of the economic development of the countries (28). For example, high-income countries like Japan and South Korea strongly relied on SCI-specialized outpatient care, while a primary-care-oriented system characterized middle-income countries like Indonesia (28). Additionally, Malaysia and Thailand demonstrated high utilization of inpatient SCI rehabilitation with hospitalization rates above the cluster average (28). High reliance on centralized SCI-specialized care presents an accessibility challenge, particularly for rural residents. PRM specialists from state hospitals regularly travel to district hospitals for consultations, potentially causing workload imbalances, especially in areas with fewer PRM specialists, such as the East Coast and East Malaysia.

Task shifting by increasing reliance on community-level healthcare workers (29) offers a potential solution to mitigate the heavy dependence on centralized SCI-specialized care. Data from 11 European countries indicated an association between stronger primary care systems and better health service access for individuals with SCI (30). During the Seventh Malaysia Plan (1996–2000), outpatient departments were relocated to district health centres to expand the primary healthcare services (31). The integration of PCPs into these health centres in 1997 mitigated unnecessary referrals and congestion in specialist clinics at hospitals by providing comprehensive health checks and treatments (31). PRM could emulate the health-delivery models of other medical specialities in Malaysia, leveraging the expertise of PRM specialists and PCPs to ensure optimal services to community-dwelling individuals with SCI (32). Sharing care between medical specialists can optimize care, aligning with the chronic disease management model, allowing the bulk of care to remain at the primary care level, and reserving specialist care for the most complex issues (32). Barriers to managing SCI at the primary care level, such as insufficient provider knowledge, lack of specialized equipment and accessible infrastructure, time constraints, and the lack of specific clinical guidelines and preventive care protocols for SCI patients (1, 3, 32–34), must be addressed. Furthermore, establishing clear roles and responsibilities between PCPs and PRM specialists, with explicit criteria for referrals to specialists and return to primary care, can improve health outcomes and reduce costs associated with suboptimal management (32). For example, Thailand’s Universal Coverage scheme allows registered PWDs to directly access higher-level hospitals without needing a referral, thereby reducing delays in managing SCI-related conditions like autonomic dysfunction (24).

The challenges of healthcare personnel shortages and uneven distribution are not unique to Malaysia. Despite its high doctor density and production, Singapore is the major importer of doctors in Southeast Asia due to shortages in the public sector (29). In Malaysia, this imbalance is particularly evident in the rural areas of East Malaysia, where there are approximately 0.5 physicians per 1,000 population, compared with the overall ratio of 0.9 in the rest of Malaysia (35). With the number of private far outstripping public healthcare facilities (6), public–private partnership (PPP) presents a promising solution (4, 5). Initiated in 2011 with the establishment of the PPP Unit in the Prime Minister’s Department, these partnerships have already yielded successes, notably in the provision of dialysis services and the expansion of medical tourism, now a key economic strategy for Malaysia (29, 36). The collaborative efforts between the public and private sectors during the COVID-19 pandemic further demonstrate the potential benefits of implementing PPP effectively (37). However, challenges such as patient referral difficulties between sectors and the need for a unified health information system must be addressed to enhance efficiency and maintain confidentiality (4). Encouraging the sharing of surplus amenities and facilitating cross-purchasing services between sectors could help bridge resource gaps and address shortages (4).

In this study, sexual dysfunction was rated the most severe health problem, aligning with a large-scale European study that highlighted sexual activity as the greatest unmet need (38). Despite concerns about bladder, bowel, and autonomic dysfunction during sexual activity, sexual health is still paramount to improving quality of life (39). However, sexual health issues are often overlooked in both inpatient and outpatient settings, particularly in Malaysia, where open discussions on the topic are rare and related literature is scarce (11). A local study found that women with SCI reported significantly lower sexual desire than able-bodied women and many delayed sexual experiences post-injury (11). The frequency of sexual activity declined after the SCI among Malaysian women, from 4.6 times to 1.5 times per month (9). Healthcare providers face barriers such as a lack of knowledge, discomfort in discussing sexual matters, and time constraints compounded by societal misconceptions about the sexuality of disabled individuals (34). Patients often expect healthcare providers to initiate discussions but feel embarrassed to raise the topic themselves (9, 40). To overcome these challenges, standardized, multidisciplinary approaches are recommended, utilizing tools such as the Permission, Limited Information, Specific Suggestions, and Intensive Therapy (P-LI-SS-IT) model and the Sexual Rehabilitation Framework to address both medical and psychosocial aspects of sexual health (40, 41). Healthcare providers should maintain an open discussion in a straightforward and non-judgemental manner and provide access to sex education in both formal and informal settings throughout the treatment continuum (41). Proactive engagement from healthcare providers across disciplines is crucial to dismantling barriers, correcting misconceptions, and fostering a supportive environment for individuals with SCI to discuss and manage their sexual health (40). Additionally, addressing other health problems, such as spasticity, contracture, and pain, is equally imperative, as these issues were significantly correlated with higher HCU amongst individuals with SCI in Malaysia.

The study participants reported high satisfaction with healthcare services, consistent with previous studies (1, 3, 24, 26). To evaluate HCSS, participants were asked about their experiences of respectful treatment, clarity of explanations from healthcare providers, involvement in treatment decisions, and overall satisfaction. Notably, Malaysia, along with the United States and Switzerland, reported the highest levels of life satisfaction among the 22 countries participating in the InSCI study (42). Despite experiencing various challenges in accessing healthcare, PWDs have been reported to be more adaptable, better at coping, and generally more satisfied with life than their able-bodied counterparts (34). However, it is essential to consider factors contributing to ALPR, identified in the path analysis as a predictor of low HCSS. A systematic review by Ginis et al. categorized over 200 facilitators and barriers to physical activity among PWDs into five levels: intrapersonal, interpersonal, institutional, community, and policy levels (43). Local researchers have identified factors across these levels, including medical issues, psychological factors, driving ability, and employment (intrapersonal); social support and attitudes (interpersonal); accessibility (institutional); facility and equipment (community); and transportation, funding, and public policy (policy) (12, 14–17, 19). However, interventions targeted at these factors still need to be improved. Ginis et al. emphasized the need to move beyond simply listing barriers and facilitators, urging the development, testing, and implementation of effective interventions. This highlights the urgent need for local interventionists to tackle factors across multiple levels and sectors to enhance activity and participation for individuals with SCI.

Interestingly, our moderation analysis revealed a gender discrepancy related to the ALPR. We observed higher ALPR in male participants with more severe SCI and health problems than their female counterparts. This finding is unprecedented in the Malaysian context, as previous studies investigating facilitators and barriers to activity and participation did not report significant gender differences (12, 14–16). One explanation for this finding could be the broader generalizability of our data compared with earlier research. The impact of SCI severity and the associated health problems on ALPR may differ between men and women in different regions of Malaysia. A significant factor to consider is the stark contrast in ethnic diversity between Peninsular Malaysia and East Malaysia, with the latter being significantly more racially and culturally diverse. Additionally, women with SCI represent a “minority within a minority” (30), making up only 21% of our study participants, which may have affected the representativeness of the findings. The role of gender in the relationship between SCI severity, health problems, and ALPR warrants further investigation.

This study has several limitations. First, the absence of a national SCI registry prevented the use of a predefined sampling frame, necessitating the reliance on convenience sampling, which may introduce self-selection bias. Additionally, although data were collected from all 5 Malaysian regions, the participating institutions were all situated in urban areas. This urban focus may limit the generalizability of the findings to the broader SCI population in Malaysia. Nevertheless, as the first multi-centre study on this topic, it provides a valuable baseline for future research. Another limitation is the lack of inter-regional comparisons. HCU and HCSS patterns may differ between the socioeconomically advanced Central region, which benefits from well-distributed rehabilitation services, and other regions with lower socioeconomic status and less accessible services. Identifying and addressing these disparities is crucial to ensuring equitable healthcare access for all individuals with SCI in Malaysia, regardless of their region of residence. It is also important to note that while path analysis, the analytical tool employed in this study, can disprove models proposing causal relations among variables, it cannot determine causality. Determining causality relies more on the study design than on the analysis itself (23). Given the cross-sectional nature of this study, it is not possible to draw causal inferences from the findings.

In conclusion, our study underscores the significant reliance on centralized specialized rehabilitation services among individuals with SCI in Malaysia and the often neglected issue of sexual dysfunction. To address these challenges, we propose decentralizing rehabilitation services by empowering primary care providers to deliver community-based care to individuals with SCI. This approach could improve accessibility and help alleviate the shortage of rehabilitation professionals. Strong collaboration between the rehabilitation and primary care sectors is essential, along with a multidisciplinary approach to providing proactive and comprehensive sexual rehabilitation care. Strengthening PPP is crucial for improving health provision and coverage. Targeted interventions that enhance facilitators and eliminate barriers to activities and participation are necessary to enhance HCSS. Achieving these goals requires a concerted effort from healthcare professionals, local interventionists, the community sector, stakeholders, and policymakers to devise effective strategies for enhancing healthcare for individuals with SCI in Malaysia.
